# LQR Control and Optimization for Trajectory Tracking of Biomimetic Robotic Fish Based on Unreal Engine

**DOI:** 10.3390/biomimetics8020236

**Published:** 2023-06-04

**Authors:** Ming Wang, Kunlun Wang, Qianchuan Zhao, Xuehan Zheng, He Gao, Junzhi Yu

**Affiliations:** 1 School of Information and Electrical Engineering, Shandong Jianzhu University, Jinan 250101, China; xclwm@sdjzu.edu.cn (M.W.); wklff654321@163.com (K.W.); zxh656@126.com (X.Z.); gaohe118@163.com (H.G.); 2 Department of Automation, Tsinghua University, Beijing 100018, China; zhaoqc@tsinghua.edu.cn; 3 Shandong Zhengchen Technology Co., Ltd., Jinan 250000, China; 4 Department of Advanced Manufacturing and Robotics, College of Engineering, Peking University, Beijing 100871, China; 5 Science and Technology on Integrated Information System Laboratory, Institute of Software, Chinese Academy of Sciences, Beijing 100190, China

**Keywords:** robotic fish, track tracking, Unreal Engine, AirSim, discrete linear quadratic regulator, particle swarm optimization

## Abstract

A realistic and visible dynamic simulation platform can significantly facilitate research on underwater robots. This paper uses the Unreal Engine to generate a scene that resembles real ocean environments, before building a visual dynamic simulation platform in conjunction with the Air-Sim system. On this basis, the trajectory tracking of a biomimetic robotic fish is simulated and assessed. More specifically, we propose a particle swarm optimization algorithm-based control strategy to optimize the discrete linear quadratic regulator controller for the trajectory tracking problem, as well as tracking and controlling discrete trajectories with misaligned time series through introducing a dynamic time warping algorithm. Simulation analyses of the biomimetic robotic fish following a straight line, a circular curve without mutation, and a four-leaf clover curve with mutation are carried out. The obtained results verify the feasibility and effectiveness of the proposed control strategy.

## 1. Introduction

As a result of the rapid growth of digital twin technology, game creation engines, such as Unreal Engine and Unity 3D, became a prominent focus of research in the construction of robot simulation systems [1,2,3]. These engines feature more realistic visual rendering performance, highly replicable dynamic physical simulation effects, and the ability to create highly depicted virtual scenes. The AirSim open-source simulation platform built using the Unreal Engine can communicate with the simulation program’s robot model using languages such as Python. This simulation benefits from a high level of input and visibility. The robot’s control system may be shown in three dimensions, dynamic models can be simulated, and tests can be verified with greater precision [4]. The utilization of Unreal Engine, Airsim, Python, etc. to construct a visual simulation platform offers a novel concept for the dynamic simulation platform of underwater robots.

In recent years, underwater robots were utilized in a variety of marine applications, including marine resource exploitation, detection of submerged pipelines, underwater rescue, and marine environment monitoring [5]. Some bionomists are developing new types of underwater robots through borrowing the movement of marine organisms [6]. Simulation technology for underwater robotics is a key enabling technology for the development and testing of underwater robots. This technology can lower the cost of testing in the actual aquatic environment, reduce the risk of testing, increase the efficiency of testing, and imitate harsh settings to deliver large reserves of data required for design and testing. Due to its compact size, low energy consumption, and rapid mobility, the trevally-like robotic fish with body/tail fin propulsion mode became the focus of research and development for underwater robots [7]. This work utilizes Unreal Engine, Airsim, and Python to establish a dynamic simulation platform for underwater robots, before conducting simulation experiments on trajectory tracking control and optimization of biomimetic robotic fish, thus providing new simulation methods and control concepts for the study of biomimetic swimming robots.

Many control approaches, such as central pattern generator, model predictive control, neural network, synovial membrane control, and adaptive robust control, were developed by domestic and international researchers for underwater robot trajectory tracking [8,9,10]. Liu et al. devised a finite-time trajectory tracking controller to assure finite-time convergence of the motion tracking error through combining the second-order sliding mode control and backstepping approach with a non-linear disturbance observer [11]. Zhang et al. designed a neural network-based adaptive controller for UUV trajectory tracking control in the presence of symmetric actuator saturation through employing neural network compensation and adaptive estimating techniques [12]. A method of adaptive control with prescribed performance was proposed to improve the effectiveness of motion control for underwater vehicles, achieving the asymptotic stability of tracking errors [13]. Integrating a central pattern generator (CPG) with sliding mode controller (SMC), Yan et al. suggested a controller based on SMC-CPG, which can rapidly reduce position and heading angle errors and achieve a stable state for the system [14]. Kong et al. combined the extended state observer and the model predictive governor to track the underactuated 3D trajectory of an underwater vehicle in a complicated and turbulent environment [15]. Heshmati et al. proposed a method of robust non-linear model predictive control that enables underwater robots to complete trajectory tracking under surge, lift, and yaw, as well as attain online trajectory planning and obstacle avoidance functions [16].

In addition, the linear quadratic regulator (LQR) was effectively applied to a variety of complicated systems, such as double inverted pendulums, fuel cell systems, vibration control systems, electric cars, and; it is also widely employed in robot trajectory tracking control [17,18,19]. The quadratic cost function of the LQR controller consists of two weighting matrices, i.e., the *Q* and *R* matrices; the *Q* weighting matrix is related to the trajectory deviation of the state variable, and the *R* weighting matrix is related to the control quantity and actuator saturation. Nonetheless, the key to creating LQR-optimized controllers for real-time applications is largely dependent on the efficient selection of *Q* and *R* weighting matrices, which necessarily involve trade-offs and are often tuned based on trial and error [20]. In addition, improving the efficiency of solving the Riccati algebraic differential equations is considered to be a progressive manifestation of designing LQR controllers [21]. Evolutionary computation (EC) was proposed as an alternative method to solve such optimization problems [22]. Deng et al. developed Bryson’s method for adjusting the *Q* and *R* weighting matrices in an effort to solve the drawbacks of the trial-and-error method [23]. Gupta et al. determined the optimal LQR weighting matrix using the non-dominated sorting genetic algorithm [24]. Elumalai et al. introduced an adaptive particle swarm optimization (APSO) technique to tackle the LQR weight optimization problem, which enhanced the system’s convergence speed [25]. Zhou et al. designed an image-guided motion controller, which consisted of a direction controller and a linear quadratic regulator (GA-LQR) speed controller based on a genetic algorithm, to achieve miniature robot swarm tracking targets through precise control of direction and speed [26]. However, the major drawback of this strategy is that there are no specific criteria for evaluating the solution’s appropriateness. Hence, the quality of the solution varies from designer to designer for identical situations utilizing the same optimization algorithm.

This paper aims to use Unreal Engine to build a dynamic visualization simulation platform for underwater robots that simulates the real ocean environment, which is extensively applied to the simulation research of biomimetic robotic fish. The main contributions of the paper are summarized as follows:

- Firstly, a visual simulation platform for underwater robots that simulates the real ocean environment is established. At present, the simulation environment developed based on the Unreal Engine is mostly employed to simulate the sky and land. This paper builds an Unreal Engine-based simulation scene of the real ocean environment and offers a dynamic and visualized underwater robot simulation platform.
- Secondly, a discrete linear quadratic regulator (DLQR) controller is designed for the biomimetic robotic fish. As the target trajectory in the simulation experiment is composed of discrete points, the LQR is discretized. Using the LQR controller, the simulation and comparison experiments that involve tracking the trajectory of the biomimetic robotic fish are carried out in three states: straight line, no-angle mutation curve, and angle mutation curve.
- Thirdly, the DLQR controller is further optimized using the PSO and DTW methods. When selecting the weighted *Q* and *R* matrices of the DLQR controller, in order to reduce the workload of testing and the influence of human factors and the local optimum, a trajectory tracking control strategy based on particle swarm optimization (PSO)-DLQR is proposed. For the time series misalignment problem of the discrete system tracking trajectory, a dynamic time warping algorithm is introduced as the performance index of PSO, which improves the convergence of the algorithm.

The remainder of this paper is organized as follows: Section 2 establishes the dynamic model of the biomimetic robotic fish, Section 3 presents the DLQR controller design process, Section 4 uses the PSO method to optimize the DLQR controller, Section 5 describes the construction steps involved in the dynamic simulation platform for underwater robots based on Unreal Engine, and Section 6 provides the concluding remarks and discusses ongoing work.

## 2. Mathematical Model of a Biomimetic Robotic Fish

**Figure 1:**
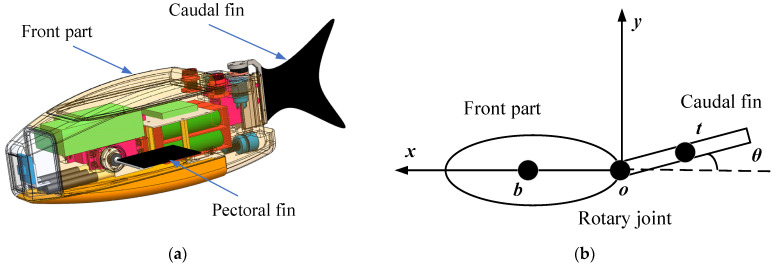
Design prototype and model of biomimetic robotic fish: (**a**) design prototype; (**b**) simplified model of robotic fish.

In this paper, a single-joint model of the biomimetic robotic fish propelled using the caudal fin is developed, as depicted in Figure 1. It is composed of two parts, i.e., a rigid front part and a swinging rear part. The rigid front part consists of a head and a portion of the body connected to the head that does not contribute to propulsion; the swing part consists of a caudal peduncle and a caudal fin. The rigid part of the biomimetic robotic fish does not generate any propulsive force. Utilizing the reaction force of water, its tail fin oscillates periodically to generate propulsive force and propels the robotic fish forward.

As illustrated in Figure 1b, the center of mass of the rigid front part of the biomimetic robotic fish is located at point *b*, and the center of mass of the swinging rear part is located at point *t*. The mass center from the rigid front part to the swinging rear part is represented by the vector $r_{bt}$, and the swing angle of the tail fin is θ.

### 2.1. Dynamic Modeling of Biomimetic Robotic Fish

Next, the force analysis is carried out from the rigid front part to the swing rear part. The dynamic model of the biomimetic robotic fish was established using the Newton–Euler formula. The hydrodynamic forces at its swinging rear are generated via additional mass forces and viscous drag. The following equation describes viscous drag.

(1) $$\left(\begin{array}{c} F_{d}\\ M_{d} \end{array}\right)=-\frac{1}{2}C\left(\begin{array}{c} \mathrm{sign}\left(V_{b}\right){\left\|V_{b}\right\|}^{2}\\ \mathrm{sign}\left(\Omega_{b}\right){\left\|\Omega_{b}\right\|}^{2} \end{array}\right)$$

The force and moment for the swinging rear can be expressed as

(2) $$\left\{\begin{array}{l} F=-m_{t}{\dot{V}}_{t}\\ M=-J_{t}{\dot{\Omega }}_{t}-\Omega_{t}\times J_{t}\Omega_{t}+r_{bt}\times F \end{array}\right.$$

The forces and moments on the rigid front can be expressed as the sum of the forces from the swinging rear and the hydrodynamic force.

(3) $$\left\{\begin{array}{l} F+F_{d}=m_{b}{\dot{V}}_{b}+m_{b}\Omega_{b}\times V_{b}\\ M+M_{d}=J_{b}{\dot{\Omega }}_{b}+\Omega_{b}\times J_{b}\Omega_{b} \end{array}\right.$$

The velocity and angular velocity of the swinging rear can be converted into the representation of the velocity and angular velocity of the rigid front.

(4) $$\left\{\begin{array}{l} V_{t}=V_{b}+\left(\Omega_{b}+\dot{\theta }e_{3}\right)\times r_{bt}\\ \Omega_{t}=\Omega_{b}+\dot{\theta }e_{3} \end{array}\right.$$

Through combining Equations (1)–(4), the kinematic parameters of the rigid front part of the biomimetic robotic fish can be expressed as

(5) $$\left\{\begin{array}{l} {\dot{V}}_{b}=\frac{F+F_{d}-m_{b}\Omega_{b}\times V_{b}}{m_{b}}\\ {\dot{\Omega }}_{b}=\frac{M+M_{d}-\Omega_{b}\times J_{b}\Omega_{b}}{J_{b}} \end{array}\right.$$

where *F* is the force on the swing rear part, *M* is the moment on the swing rear part, $F_{d}$ is the viscous drag produced using the swing rear part swinging through the water, $M_{d}$ is the viscous drag moment, *C* is the viscous coefficient, $m_{b}$ is the mass of the rigid front part, $m_{t}$ is the mass of the swing rear part, $V_{b}$ is the velocity of the rigid front part, $\Omega_{b}$ is the angular velocity of the rigid front part, $V_{t}$ is the velocity of the swing rear part, $\Omega_{t}$ is the angular velocity of the swing rear part, and $e_{3}=[0,0,1{]}^{T}$.

### 2.2. Path Tracking Error Model

The path followed by the biomimetic robotic fish in this paper is in a horizontal plane with a fixed depth; thus, the motion error model of the biomimetic robotic fish in the world coordinate system only considers the two-dimensional case. Consequently, only the yaw angle φ needs to be considered when calculating the attitude angle. The position and posture error $p_{e}$, which is located between the actual position and posture $p_{c}=(x_{c},y_{c},\varphi_{c}{)}^{T}$ and the desired position and posture $p_{r}=(x_{r},y_{r},\varphi_{r}{)}^{T},$ can then be expressed as

(6) $$p_{e}=\left(\begin{array}{c} x_{e}\\ y_{e}\\ z_{e} \end{array}\right)=\left(\begin{array}{ccc} \cos\varphi_{r} & -\sin\varphi_{r} & 0\\ \sin\varphi_{r} & \cos\varphi_{r} & 0\\ 0 & 0 & 1 \end{array}\right)\left(\begin{array}{c} x_{r}-x_{c}\\ y_{r}-y_{c}\\ z_{r}-z_{c} \end{array}\right)$$

## 3. Design of DLQR Controller

The LQR controller is employed to solve a biomimetic fish’s trajectory tracking problem, which, in turn, solves a continuous system of state quantities to track a reference trajectory. However, in general, the target trajectory is a discrete trajectory composed of discrete points; thus, the LQR controller is discretized to obtain a DLQR controller. The state space equations of the discrete control system can be expressed as

(7) $$x\left(k+1\right)=Ax\left(k\right)+Bu\left(k\right)$$

The purpose of the DLQR controller is to make the biomimetic fish trajectory tracking state error as small as possible while maintaining the input of the control quantity as small as possible to ensure the control stability. Thus, the objective function is defined as follows:

(8) $$J=\frac{1}{2}\sum_{k=k_{0}}^{\infty }x^{T}\left(k\right)Qx\left(k\right)+u^{T}\left(k\right)Ru\left(k\right)$$

where *Q* is a semi-positive definite matrix that constrains the state cost of the system, *R* is a positive definite matrix that constrains the input cost of the system, and the control law which yields the best response is shown as follows:

(9) $$u=-Kx\left(t\right)$$

In order to stabilize the control system, a constant matrix *P* is designed, and the optimal state feedback matrix *K* can be obtained as follows:

(10) $$K={\left(R+B^{T}PB\right)}^{-1}B^{T}PA$$

where *P* is the solution of the Riccati algebraic differential equation.

(11) $$P=-A^{T}PB{\left(R+B^{T}PB\right)}^{-1}B^{T}PA+A^{T}PA+Q$$

## 4. PSO-Based Optimization of DLQR Controller

When using the DLQR controller to control the robot’s trajectory tracking, the tracking performance’s evaluation index is determined based on the weighting matrices *Q* and *R*. Therefore, selecting a suitable weighting matrix parameter is the key to controlling the trajectory tracking. In the previous parameter selection process, the parameters of *Q* and *R* matrices are usually obtained via experimental or trial-and-error methods and summarized according to personal experience, which is often not the optimal solution. To achieve more accurate control of robot trajectory tracking, this paper introduces the Particle Swarm Optimization (PSO) algorithm to optimize the parameter selection of *Q* and *R* matrices, which improves the trajectory tracking control performance of traditional DLQR controllers to a certain extent.

In the design process, the desired trajectory and the actual trajectory calculated using the DLQR controller are trajectories composed of discrete points. The errors of the corresponding points are significant and inevitable because their time series are not aligned; thus, the objective optimization function of DLQR cannot be directly used as the fitness function of the PSO optimization algorithm. Since trajectory tracking mainly evaluates the overlapping similarity of two trajectories, a common approach is to use the Euclidean distance to calculate the error distance of two trajectories. However, a growing number of studies show that Euclidean distance has serious drawbacks in measuring the similarity of time series. These drawbacks mainly lie in the fact that if two sequences are only unaligned at data points, while the trajectories are very close, the calculated Euclidean distance error will still be very large, which will not accurately measure the similarity of the two sequences. The Dynamic Time Warping (DTW) algorithm can effectively solve the above problem via a very clever dynamic planning method to achieve the dynamic regularization of the time axis, meaning that all data points have the best alignment [27]. The algorithm focuses on the shape of the time series and eliminates the errors caused by the unaligned data points, thus obtaining more accurate distance values.

The DTW algorithm is defined as follows. Given a target sequence $X=(x_{1},x_{2},\cdots x_{i})$ and a test sequence $Y=(y_{1},y_{2},\cdots y_{j})$, the function $Dist(i,j)=f(x_{i},y_{j})$ is the Euclidean distance from the point to the point of the corresponding sequence, and the distance matrix *D* is constructed for the sequences *X* and *Y*. Based on the constructed distance matrix *D*, a path is found from the upper left corner to the lower right corner such that the sum of the element values through which the route passes is minimized. Next, according to monotonicity and constraint, the previous point of (i,j) can only be one of the three points on (i−1,j), i−1,j−1), and (i,j−1) is one of the three points. Thus, the formula to calculate the distance becomes

(12) $$DTW\left(i,j\right)=Dist\left(i,j\right)+\min\left[DTW\left(i-1,j\right),DTW\left(i,j-1\right),DTW\left(i-1,j-1\right)\right]$$

where DTW(i,j) denotes the cumulative distance between the two trajectories, and Dist(i,j) indicates the Euclidean distance between the *i*-th and *j*-th points of the two sequences.

The algorithm calculates the sum of the distances of errors between two trajectories to measure the similarity between two time series trajectories and use it as the fitness function of the PSO algorithm. Compared with using the controller’s objective function to calculate the fitness value of the optimization algorithm, the computational efficiency of PSO is improved to a certain extent, thus effectively improving the convergence.

The position and velocity of the biomimetic fish are set as the state quantities $x={\left(x_{e},y_{e},{\dot{x}}_{e},{\dot{x}}_{e}\right)}^{T},$ and the acceleration $u=\left({\ddot{x}}_{e},{\ddot{y}}_{e}\right)$ is set as the control quantity and brought into the state space Equation (7) of the bulk control system, where

$$A=\left[\begin{array}{cccc} 1 & 0 & \Delta T & 0\\ 0 & 1 & 0 & \Delta T\\ 0 & 0 & 1 & 0\\ 0 & 0 & 0 & 1 \end{array}\right],\;B=\left[\begin{array}{cc} 0 & 0\\ 0 & 0\\ \Delta T & 0\\ 0 & \Delta T \end{array}\right]$$

The initial *Q* and *R* matrices are set as unit matrices, and the desired acceleration *u* is calculated.

**Figure 2:**
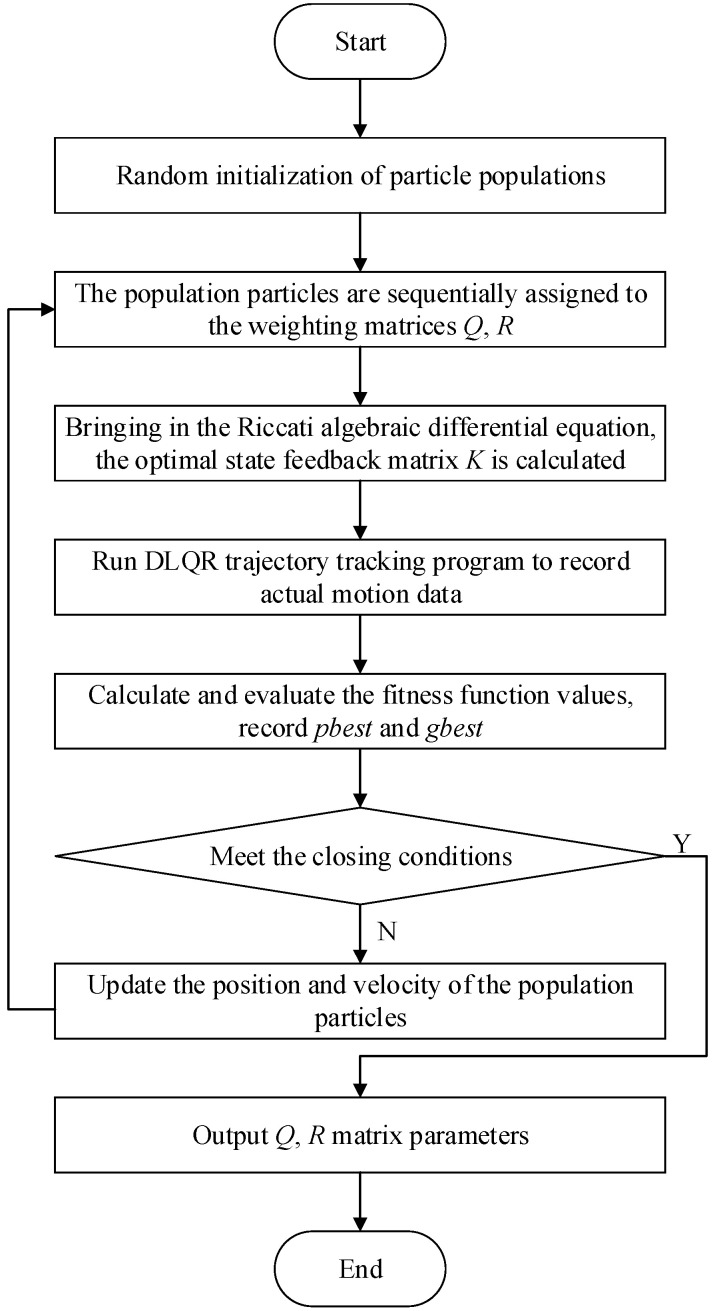
Flow chart explaining how to use PSO algorithm for optimization of weighting matrix.

The weighting matrices *Q* and *R* are optimized using the particle swarm optimization algorithm, and the optimization flow chart is shown in Figure 2.

The specific optimization steps of the particle swarm optimization algorithm are as follows:

- Step 1: Particle population initialization: we initialize the number of populations as *N* = 10, the feasible solution dimension as *D* = 6, and the maximum number of iterations as 100. Moreover, the *Q* matrix parameters are restricted to $Q_{limit}=[1,10]$, the *R* matrix parameters are limited to $R_{limit}=[0.1,1]$, the inertia weight is *w* = 0.7298, the cognitive learning factor is $c_{1}=1.497$, and the social learning factor is $c_{2}=1.497$.
- Step 2: The particle population individual $P=[Q_{1},Q_{2},Q_{3},Q_{4},R_{1},R_{2}]$, which is, in turn, assigned to *Q* and *R* matrices, is brought into the Riccati algebraic differential equation to calculate the optimal state feedback matrix *K*. The trajectory tracking program of the biomimetic machine fish is run to correctly calculate and record the coordinate data points of the actual motion trajectory. The reference and actual trajectory are brought into Equation (11) to calculate and evaluate the fitness function values iteratively, and the optimal historical position *pbest* and the optimal global position *gbest* of the particle population are obtained.
- Step 3: If the terminal condition is satisfied, we output the global optimal *Q* and *R* matrix parameters and end the program. Otherwise, we continue the execution.
- Step 4: We update the velocity and position of the particle and turn to Step 2 to continue the execution. According to the optimal historical position and the optimal global position of the particle, the velocity and position are updated using Equations (13) and (14).

(13) $$v_{i}^{t+1}=wv_{i}^{t}+c_{1}r_{1}\left(pbest_{i}^{t}-x_{i}^{t}\right)+c_{2}r_{2}\left(gbest_{i}^{t}-x_{i}^{t}\right)$$

(14) $$x_{i}^{t+1}=x_{i}^{t}+v_{i}^{t+1}$$

where $v_{i}^{t+1}$ and $x_{i}^{t+1}$ denote the velocity and position of the *i* particle at time slot *t* + 1, and $r_{1}$ and $r_{2}$ are random numbers in the interval [0, 1].

The fitness function *E* is expressed as follows:

(15) E=DTW

The optimized weighting matrix is

$$Q_{op}=\left[\begin{array}{cccc} 10 & 0 & 0 & 0\\ 0 & 10 & 0 & 0\\ 0 & 0 & 1 & 0\\ 0 & 0 & 0 & 1 \end{array}\right]\;\mathrm{and}\;R_{op}=\left[\begin{array}{cc} 0.8 & 0\\ 0 & 1 \end{array}\right]$$

## 5. Establishment of UE-Based Simulation Platform

The simulation environments developed via traditional robot simulation platforms, such as Gazebo, Webots, and CoppeliaSim (formerly known as V-REP), have multiple problems, such as simpler design and lack of ability, which make it difficult to build large and complex scenarios under guaranteed simulation realism [28,29,30]. To solve the simulation platform environment modeling ability shortage and improve the simulation effect’s visualization, this paper builds a simulation environment based on Unreal Engine and AirSim to simulate dynamic ocean for underwater robotics. Due to its realistic visual rendering performance, effects that assist dynamic physical simulation, and robust data interfaces, Unreal Engine facilitates the environment simulation necessary for scientific study. AirSim is an open-source simulation environment designed specifically for unmanned robots. It became a simulation platform for marine settings via transforming simulation models and simulation environments.

The specific steps required to establish a UE-based ocean simulation environment are as follows:

- Step 1: Build an ambient light source. Add “Directional Light”, “Sky Light”, and “Visual Effects” from “Light Sources” to the “Sky Atmosphere”, “Volume Clouds”, and “Exponential Height Fog” in the viewport area. In order to simulate effects such as ambient light effect and refraction and reflection of the water surface, materials and writing scripts should be complemented.
- Step 2: Build the undersea landscape. In the “Landscape Mode”, draw the undersea landscape, add surface materials and undersea obstacles, and add some underwater elements to enrich the scene, such as seaweed, rocks, shipwrecks, etc.
- Step 3: Add the ocean water body. Add the “Water” plug-in in “Selection Mode”, and adjust the land and seafloor shape, curve, etc.
- Step 4: Simulate ocean waves. According to the Gerstner Wave formula, the offset value and normal value are calculated to simulate the effect of sharp crests of water waves. The Gerstner Wave calculation formula can be expressed as

(16) $$P\left(x,y,t\right)=\left(\begin{array}{c} x+\sum \left(Q_{i}A_{i}\times D_{i}\cdot x\times \cos\left(\omega_{i}D_{i}\cdot \left(x,y\right)+\varphi_{i}t\right)\right)\\ y+\sum \left(Q_{i}A_{i}\times D_{i}\cdot y\times \cos\left(\omega_{i}D_{i}\cdot \left(x,y\right)+\varphi_{i}t\right)\right)\\ \sum \left(A_{i}\sin\left(\omega_{i}D_{i}\cdot \left(x,y\right)+\varphi_{i}t\right)\right) \end{array}\right)$$

where $Q_{i}$ is the is the parameter that controls the steepness of the wave; $A_{i}$ is the amplitude, which represents the range of water wave fluctuations; $D_{i}$ is the wave vector, which represents the direction of the water wave moving in the plane; $\omega_{i}$ represents the angular frequency, and the wavelength can be calculated from $\lambda_{i}=2\pi /\omega_{i}$; and $\varphi_{i}$ is the phase difference, which represents the offset of the water wave in the direction of a component.

- Step 5: Add the biomimetic robotic fish model and associate the model with AirSim. The control script is written in Python to run and call the control interface of the AirSim platform for real-time dynamic control simulation implementation in the ocean scene edited using Unreal Engine.

**Figure 3:**
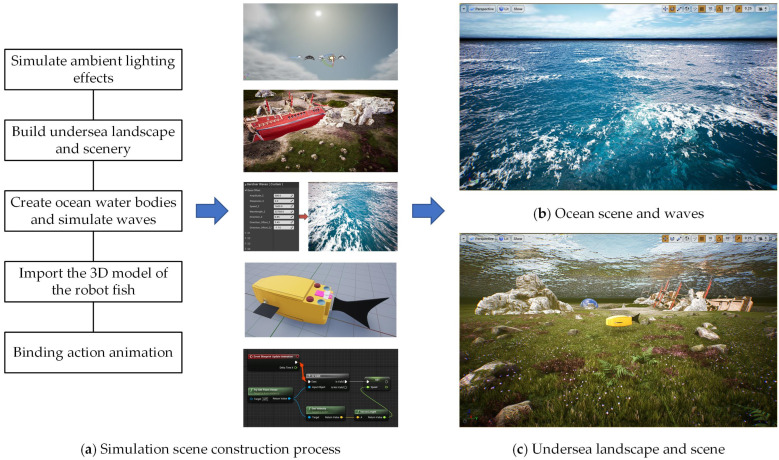
Ocean simulation environment realization process and effect.

The establishment process and the effect of the established UE-based dynamic visualization simulation platform are shown in Figure 3. Among them, (a) shows the process of establishing the simulation environment and the corresponding realized scenes, (b) indicates the realized sea surface and wave scenes, and (c) indicates the realized subsea scenes.

## 6. Results and Discussion

Based on the built simulation environment, the trajectory tracking control simulation of the biomimetic robotic fish is performed before and after optimizing the weighting matrix parameters of the DLQR controller to track a straight line, a circular curve without angular mutation, and a four-leaf clover curve with angular mutation trajectory, respectively. In the simulation diagram, the solid green line is the reference trajectory, and the solid red line is the tracking trajectory. In the trajectory diagram, the black line is the reference trajectory, the red line is the DLQR-controlled tracking trajectory, and the green line is the PSO-optimized controlled tracking trajectory.

### 6.1. Tracking a Straight Line

**Figure 4:**
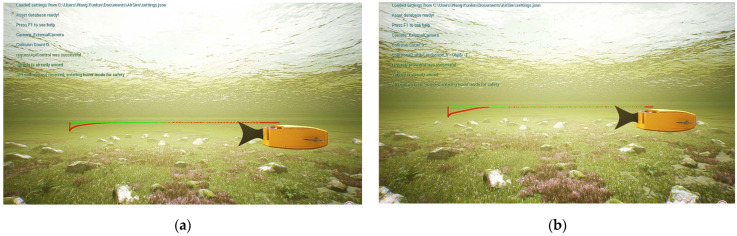
Tracking results of a straight line trajectory: (**a**) results with a straight line without optimization; (**b**) optimal results with a straight line.

**Figure 5:**
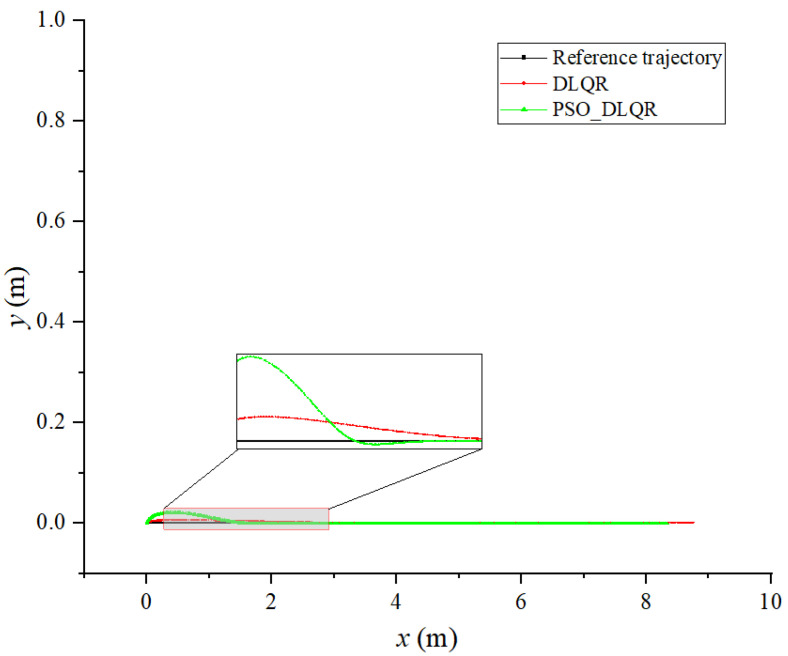
Comparison of tracking results for a straight trajectory.

In the process of tracking the straight line trajectory of the biomimetic robotic fish, the sum of the distance error between the reference trajectory and the tracking trajectory before the optimization of the weighting matrix obtained using the dynamic time warping algorithm is $DTW_{no}=33.403$ m, while after the optimization, it is $DTW_{op}=10.321$ m. Moreover, the average distance error of the corresponding points of the trajectory before the optimization is $Dist_{no}=0.042$ m, while after the optimization, it is $Dist_{op}=0.013$ m. According to Figure 4 and Figure 5, the robotic fish can follow the target trajectory faster after the parameter optimization, while the overall error of the tracking trajectory and the lag of the tracking trajectory are smaller. The DLQR controller has good control effect on the robot’s straight line tracking trajectory.

### 6.2. Tracking a Circular Curved Trajectory

**Figure 6:**
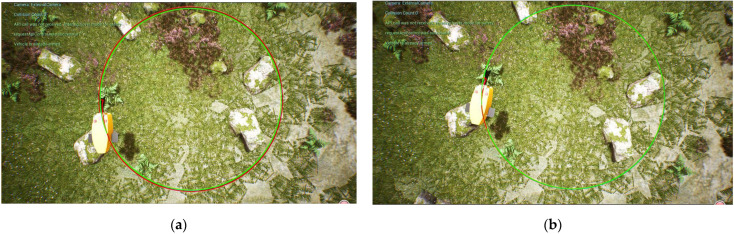
Tracking results of a circular trajectory: (**a**) results with a circular trajectory without optimization; (**b**) optimal results with a circular trajectory.

**Figure 7:**
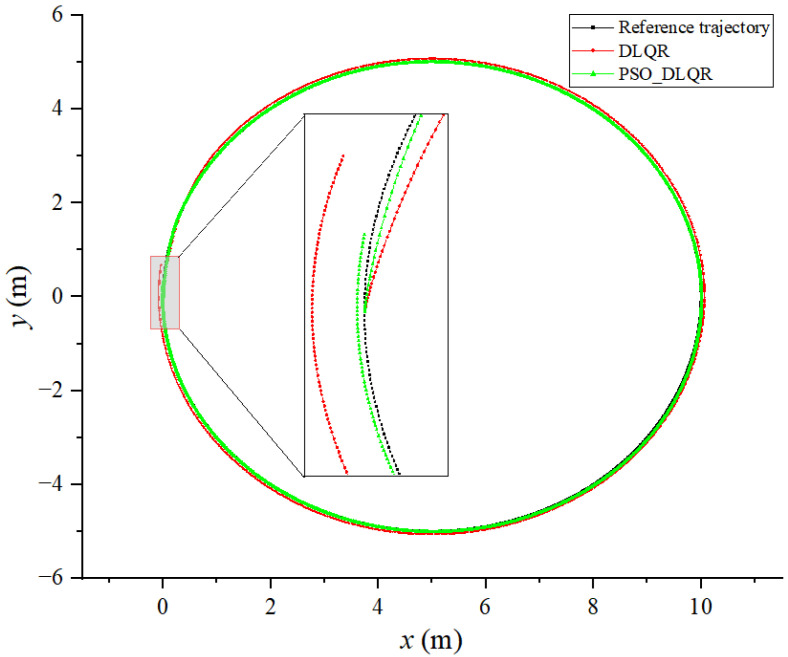
Comparison of tracking results for a circular trajectory.

According to the dynamic time warping algorithm, the sum of the distance error between the target trajectory and the tracking trajectory before the optimization of the weighting matrix is $DTW_{no}=124.183\;$ m, while after the optimization, it is $DTW_{op}=23.586\;$ m. The average distance error between the corresponding points of the trajectory before the optimization is $Dist_{no}=0.155$ m, while after the optimization, it is $Dist_{op}=0.029$ m. From Figure 6 and Figure 7, it can be observed that the DLQR control before and after the optimization of the weighting matrix can better control the machine fish’s tracking circular curve trajectory, while the offset of the tracking trajectory in the *y* direction is smaller because there is no abrupt change in the angle of the circular curve. However, the tracking trajectory of the DLQR controller before the parameter optimization has a large lag; thus, it leads to a larger tracking error.

### 6.3. Tracking a Four-Leaf Clover Trajectory

**Figure 8:**
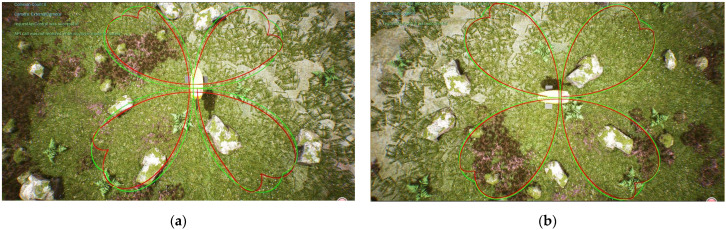
Tracking results of biomimetic robotic fish with a four-leaf clover trajectory: (**a**) results with a four-leaf clover trajectory without optimization; (**b**) optimal results with a four-leaf clover trajectory.

**Figure 9:**
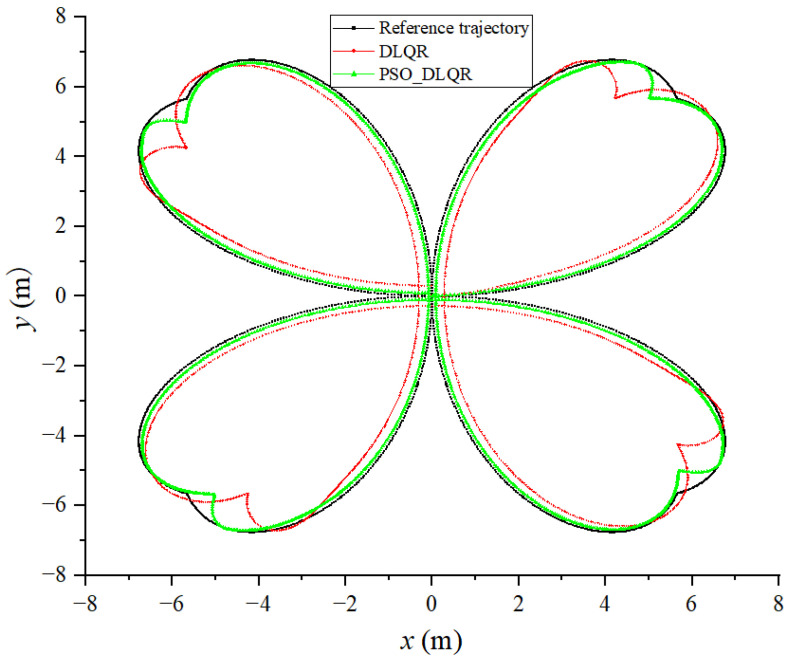
Comparison of tracking results for a four-leaf clover trajectory.

The sum of the distance errors between the reference trajectory and the tracking trajectory before the optimization of the weighting matrix obtained using the dynamic time warping algorithm is $DTW_{no}=479.332$ m, while after the optimization, it is $DTW_{op}=180.515\;$ m. The average distance error between the corresponding points of the trajectory before the optimization is $Dist_{no}=0.299$ m, while after the optimization, it is $Dist_{op}=0.112$ m. Observing Figure 8 and Figure 9, it becomes evident that the biomimetic machine fish in the tracking curve trajectory of the sudden change is at an angle. Due to the lag affecting the controller, the tracking trajectory produced a significant deviation;m thus, the average error distance for the tracking trajectory is larger. The DLQR controller with optimized parameters of PSO can adjust the control object to follow the reference trajectory faster. The DLQR controller with initial parameters, on the other hand, is less effective in the process of tracking curved trajectories with abrupt angular changes.

### 6.4. Discussion

Synthesizing the above trajectory tracking testing results, we found that the Unreal Engine-based ocean simulation environment can make the experimental process more realistic and intuitive, and can effectively observe the dynamic changes of the environment and the real response of the experimental object during the experiment, which can effectively improve the dynamic simulation experiment effect. The test results indicate that the DLQR controller with initial *Q* and *R* weighting matrix parameters can track the target trajectory more quickly when using the biomimetic robotic fish to track straight and curved trajectory without abrupt angle changes; however, the tracking lag is larger. When tracking the curved trajectory with abrupt angle changes, it is difficult to adjust the control attitude quickly to track the target trajectory under the continuous large angle changes due to the large tracking lag, while the tracking effect is poor. The DLQR controller with optimized *Q* and *R* weighting matrix parameters based on the PSO algorithm can track the target trajectory quickly and with more limited tracking errors when using the biomimetic robotic fish to track straight trajectory and curve trajectory without abrupt angle changes. When tracking the curve trajectory with abrupt angle changes, the trajectory deviates due to the hysteresis of the tracking; however, it can still adjust the control attitude quickly and track the target trajectory.

To further analyze the control effectiveness of the DLQR method, we compared it to the model predictive control method. At each sampling moment, the model predictive controller’s current control action is determined through solving a finite time domain open-loop optimal control problem. The current state of the process is viewed as the starting point for the optimal control problem, and the optimal control sequence found only implements the first control action. In Equation (17), we allow the robotic fish’s position and velocity to be state variables, and allow the acceleration to be control input variables.

(17) $$x=\left[\begin{array}{l} p_{x}\\ p_{y}\\ v_{x}\\ v_{y} \end{array}\right],\;u=\left[\begin{array}{l} a_{x}\\ a_{y} \end{array}\right]$$

Therefore, the state equation of the system can be expressed as

(18) $$\dot{x}(t)=\tilde{A}x(t)+\tilde{B}u(t)$$

When $\dot{x}=\frac{x(k+1)+x(k)}{\Delta T}$, the following formula can be obtained via discretizing the above system.

(19) $$x(k+1)=(I+\Delta T\tilde{A})x(k)+\Delta T\tilde{B}u(k)$$

If we assume that $A=(I+\Delta T\tilde{A})$, $B=\Delta T\tilde{B}$, it is also true that

(20) x(k+1)=Ax(k)+Bu(k)

where $A=\left[\begin{array}{cccc} 1 & 0 & \Delta T & 0\\ 0 & 1 & 0 & \Delta T\\ 0 & 0 & 1 & 0\\ 0 & 0 & 0 & 1 \end{array}\right]$ and $B=\left[\begin{array}{cc} 0 & 0\\ 0 & 0\\ \Delta T & 0\\ 0 & \Delta T \end{array}\right]$.

**Figure 10:**
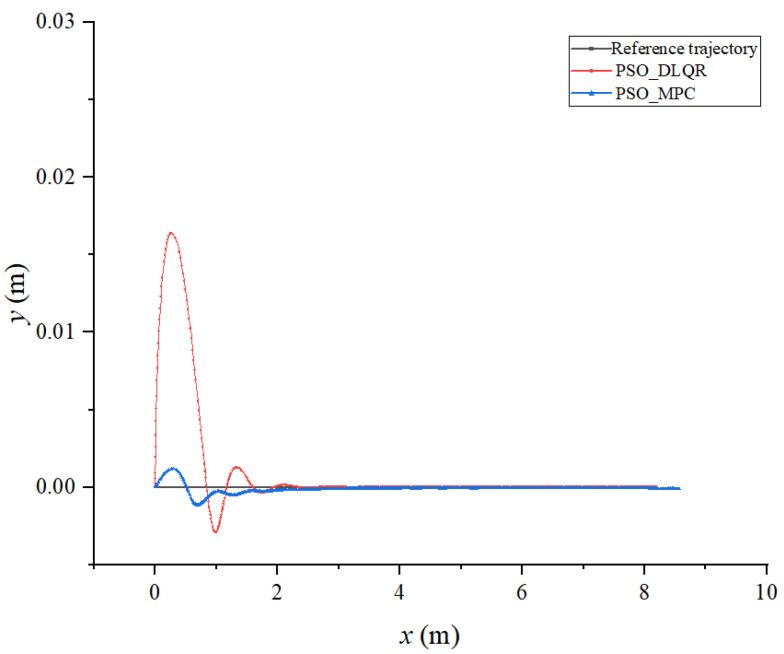
Results of a line trajectory using both methods.

**Figure 11:**
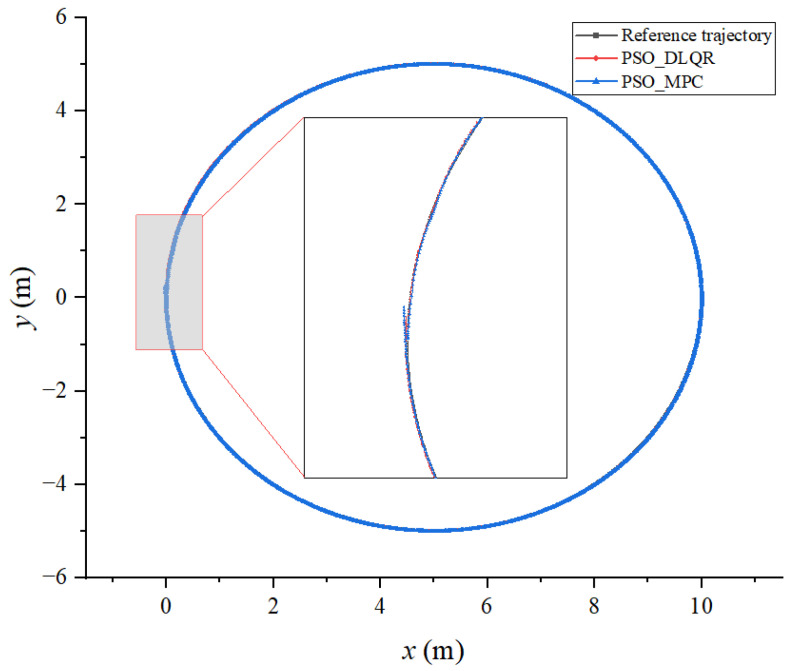
Results of a circle trajectory using both methods.

**Figure 12:**
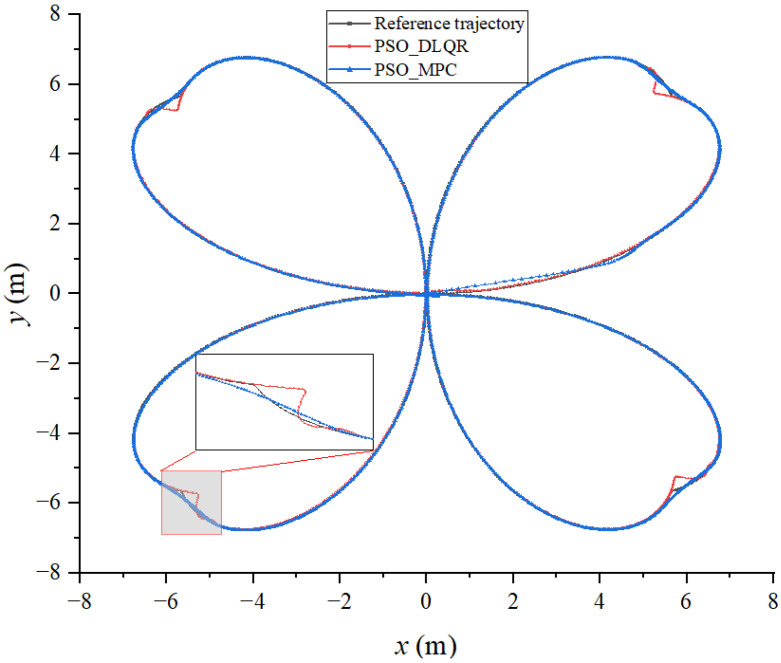
Results of a four-leaf clover trajectory using both methods.

The biomimetic robotic fish is then controlled using the model predictive control approach, which is also optimized using PSO algorithm, to track the reference trajectories, which are a straight line, a circle, and a four-leaf clover-shaped curve. The DTW distance and average DTW of trajectory tracked through the optimized DLQR method and the optimized MPC method are shown in Table 1, Table 2 and Table 3. The trajectories of the bionic robotic fish determined using the two methods are shown in Figure 10, Figure 11 and Figure 12. According to the obtained results, the optimized DLQR approach outperforms the optimized MPC method in line tracking. However, there will be some latency when the reference trajectory abruptly changes. While the improved MPC technique performs better on curve tracking, it cannot properly reconstruct the shape of curves when an abrupt change occurs.

## 7. Conclusions

This paper develops an Unreal Engine-based framework for dynamic simulation of real ocean environments. It integrates the AirSim simulation system to visualize the trajectory tracking control of the biomimetic robotic fish and observe the motion state of the biomimetic robotic fish in real time as it swims in the ocean environment, resulting in a more realistic simulation effect and a higher degree of visualization. On this dynamic simulation platform, we propose a control strategy based on the PSO algorithm to optimize the DLQR controller for the trajectory tracking problem of the biomimetic robotic fish, as well as to track and control discrete trajectories with misaligned time series through introducing the dynamic time warping algorithm. After employing PSO to optimize the *Q* and *R* weighting matrices, the simulation results indicate that the DLQR controller’s accuracy can be effectively enhanced.

Although this paper gives some fascinating simulated examples of trajectory tracking of biomimetic robotic fish under perfect settings, it does not take into account aquatic environmental influences, and the control model design is also relatively simplistic. To further investigate the dynamic control mechanism of the biomimetic robotic fish operated in ocean scenarios, we will increase the complexity of the control model and further explore the impact of environmental changes and obstacles in future work.

## Figures and Tables

**Table 1:** Results of tracking a straight line using both methods.

Tracking Methods	PSO_DLQR	PSO_MPC
DTW distance (m)	6.027	15.054
Average DTW (m)	0.007	0.019

**Table 2:** Results of tracking a circle using both methods.

Tracking Methods	PSO_DLQR	PSO_MPC
DTW distance (m)	18.147	15.684
Average DTW (m)	0.011	0.001

**Table 3:** Results of tracking a four-leaf clover-shaped curve using both methods.

Tracking Methods	PSO_DLQR	PSO_MPC
DTW distance (m)	65.009	49.129
Average DTW (m)	0.041	0.031

## Data Availability

The data generated during the current study are available from the corresponding author on reasonable request.
